# Hypertension, diabetes, and cardiovascular disease nexus: investigating the role of urbanization and lifestyle in Cabo Verde

**DOI:** 10.1080/16549716.2024.2414524

**Published:** 2024-10-22

**Authors:** Aaron Kobina Christian, Akosua Afriyie Osei-Appaw, Ruth Tobi Sawyerr, Martin Wiredu Agyekum

**Affiliations:** a Regional Institute for Population Studies, University of Ghana, Accra, Ghana; b Institute for Educational Research and Innovation Studies (IERIS), University of Education, Winneba, Ghana

**Keywords:** Cardiovascular diseases, hypertension, diabetes, lifestyle modifications, developing countries

## Abstract

**Background:**

Although hypertension and diabetes are known to increase cardiovascular disease risk, the influencing and underlying factors remain unclear.

**Objective:**

To examine the mediating effect of location of residence and the moderating effects of recommended lifestyle practices in the hypertension/diabetes and cardiovascular disease nexus.

**Material and methods:**

Data were drawn from 4,563 participants in Steps 1 and 2 of the 2020 World Health Organization’s STEPS survey in Cabo Verde, with a subsample of 2,436 individuals completing Step 3. A logit regression model was employed to examine the correlations of cardiovascular disease, while structural equation modeling and interaction analysis were used to identify mediators and moderators within the explored relationships.

**Results:**

Living with hypertension and diabetes were both observed to increase the likelihood of having a cardiovascular disease [Coeff. (RSE), 0.46, (0.12), *p* < 0.001; 1.26, (0.14), *p* < 0.001, respectively]. We identify urbanicity as a potential mediator through which hypertension/diabetes leads to a cardiovascular disease and waist circumference as a moderator of hypertension/diabetes-cardiovascular nexus.

**Conclusion:**

These findings add to the toolset of public health practitioners and policymakers in formulating policies and interventions aimed at managing cardiovascular diseases, particularly in developing nations.

## Paper context

- **Main findings**: The nexus between hypertension, diabetes, and cardiovascular diseases is mediated and moderated by urbanicity and waist circumference, respectively.
- **Added knowledge**: The effect of the place of residence or location on disease proclivity is contextual. The urban environment may have varying effects on non-communicable disease incidence.
- **Global health impact for policy and action**: Addressing the management of cardiovascular disease incidence demands a holistic strategy, encompassing a proper understanding of location dynamics, as well as the role recommended lifestyle modification plays.

## Background

Globally, non-communicable diseases (NCDs) are responsible for approximately three-quarters of all annual deaths [1]. The burden of NCDs in sub-Saharan Africa (SSA) keeps rising and can be attributed partly to the region’s current epidemiology and nutrition transitions [2]. While approximately a quarter of the population in SSA suffered from NCDs in 2004, this is projected to rise to nearly half of the population (specifically, 46%) by 2030 [3]. Although there are several factors accounting for the rise in NCDs, urbanization is noted to have contributed significantly to this rise [4]. Urbanization is often linked to decreased physical activity, consumption of unhealthy foods, and increased smoking and alcohol consumption [5,6].

Among NCDs, cardiovascular diseases (CVD) take the lead, with ischemic heart disease (IHD) emerging as the predominant cause of cardiovascular-related mortality [7]. The age-adjusted CVD mortality rate for high-income countries (HICs) is higher than that of SSA; however, the latter has witnessed more than a 50% increase in CVD-related deaths over the past three decades [8] with a significant amount of these deaths occurring in younger adults [9–11]. The two most prevalent NCDs of public health concern are diabetes and hypertension [12]. Although the etiologies linking both diabetes and hypertension remain speculative, several epidemiologic studies provide evidence for their co-existence [13].

The incidence of hypertension and diabetes, along with their contribution to CVD, may vary by location and be influenced by individual lifestyle behaviors. A critical gap requiring further investigation is the need for a deeper understanding of the potential mediating and moderating factors in these relationships. Such understanding of these connections is pivotal in devising more effective preventive strategies, improving the management of co-morbid conditions, and ultimately, fostering advancements that can lead to saving lives. Drawing on the Social Determinants of Health (SDoH) framework [14], this study examines how social, economic, and environmental factors interact to influence health outcomes, particularly cardiovascular disease (CVD). The tenets of this theory play a crucial role in shaping lifestyle choices thus contributing to the development of CVD. Consequently, this study seeks to answer the following questions: 1) Does hypertension and diabetes affect the incidence of CVD in Cabo Verde? 2) If it does, what are the channels through which this occurs? and 3) do recommended lifestyle modifications moderate the effect of the relationship between hypertension, diabetes, and CVD?

## The relationship between hypertension/diabetes and CVD

Some observational studies have demonstrated that hypertension and diabetes are significant contributors to the incidence of CVD [15–18]. Concisely, elevated blood pressure damages the endothelium, leading to atherosclerosis, arterial stiffening, and increased risk of cardiovascular events like heart attacks and strokes. Type 2 diabetes, on the other hand, contributes to CVD through insulin resistance, dyslipidemia, hyperglycemia, and endothelial dysfunction. These factors promote atherosclerosis, inflammation, and blood vessel damage, further elevating CVD risk. Thus, first, we hypothesize that individuals who have either hypertension or diabetes are more likely to develop CVD in Cabo Verde.

H1: Living with hypertension or diabetes increases the likelihood of an individual having a CVD.

The dialogue on NCDs is incomplete without discussing the role location plays in this conundrum. Urban residents with hypertension or diabetes are often exposed to risk factors such as prolonged working and sitting hours, high-cholesterol diets, and air and noise pollution. Conversely, rural residents adopting urban lifestyle patterns, combined with limited access to health information and infrastructure, may face increased risks of cardiovascular diseases. Considering the various location-related factors that can influence the link between hypertension, diabetes, and cardiovascular disease (CVD), we hypothesize that where you live plays a role in mediating this connection.

H2: Where you live, as in a rural or urban area, mediates the association between hypertension/diabetes and the incidence of CVD.

The WHO recommends a healthy diet, regular physical activity, limiting alcohol, and avoiding tobacco to reduce NCD risk. Although these lifestyle changes have shown potential in reducing non-communicable diseases (NCDs) and their effects, there is insufficient evidence to confirm their effectiveness in lowering cardiovascular disease incidence among individuals with hypertension or diabetes. This leads to our third hypothesis:

H3: Adherence to recommended lifestyle choices moderates the effect of hypertension/diabetes on CVD incidence.

**Figure 1.**
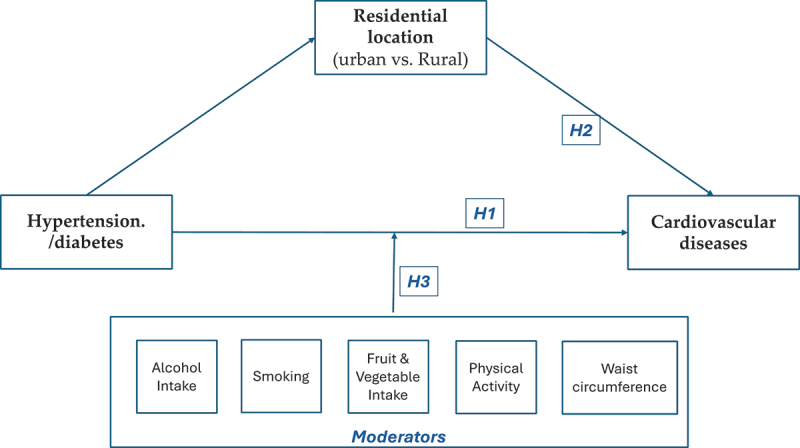
Analytical framework for the hypertension/diabetes -CVD nexus.

Figure 1 presents an analytical framework that examines the relationship between hypertension/diabetes and CVD, the moderators, and mediators as highlighted in the literature reviewed above.

## Methodology

### Study design and data source

The dataset for this study was obtained during the second World Health Organization (WHO) STEPS cross-sectional survey on noncommunicable disease (NCD) risk factors in Cabo Verde, conducted from February to March 2020. The survey was structured into three sequential steps: Step 1 involved the collection of socio-demographic and behavioral information. Step 2 was dedicated to acquiring physical measurements, encompassing parameters such as height, weight, and blood pressure. In Step 3, blood and urine samples were collected to conduct biochemical measurements, evaluating factors such as blood glucose levels, cholesterol levels, and salt intake. The STEPS data were suitable for this study due to the use of standardized methods for data collection with attention to prevalence and risk factor analysis.

### Population and sampling

The survey targeted a population of adults aged 18 to 69 in Cabo Verde. To ensure the representativeness of the data within this age range, a multiple-stage probability sampling design was employed. A total of 4,563 adults participated in Steps 1 and 2, and a subsample of 2,436 individuals took part in Step 3. The overall response rate for the survey was calculated to be 64%, indicating the proportion of individuals who actively participated in the study. The study was authorized by the Data Protection Commission (CNPD) and the National Ethics Committee for Health Research (CNEPS), Cabo Verde. All participants provided written informed permission.

## Measures

### Outcome and main explanatory variables

The outcome variable of interest was having a CVD. Individuals who report having had a heart attack or chest pain from heart disease (angina) or a stroke take aspirin regularly to prevent or treat heart disease or are currently taking statins (Lovastatin, Simvastatin, Atorvastatin, or any other statin) regularly to prevent or treat heart disease.

The two main independent or explanatory variables were hypertension and diabetes. An individual was described as living with hypertension when his/her systolic blood pressure (SBP) ≥140 mm Hg, or a diastolic blood pressure (DBP) ≥90 mm Hg, or uses medications intended to lower blood pressure for the management of hypertension. Persons with a fasting capillary blood glucose level equal to or exceeding 126 mg/dL, or those using medications to control elevated blood sugar, were identified as having diabetes mellitus.

### Mediating and moderating variables

The mediating variable of interest was the residential location. Respondents were living either in an urban area or rural area. Recommended lifestyle modifications such as avoidance of smoking, reduction of alcohol intake, and intake of fruits and vegetables were explored as the possible moderators of hypertension/diabetes-CVD nexus. Respondents who had smoked within the past 30 days were categorized as current smokers. Harmful use of alcohol was defined as consuming 60 grams of pure alcohol on an average day in the past 30 days, or a man having 4 or more and a woman having 3 or more standard drinks per week, respectively. Insufficient fruit and vegetable intake was identified when participants consumed fewer than five servings of fruits and vegetables per day [19]. Insufficient physical activity was determined for individuals engaging in less than the equivalent of 150 minutes of moderate-intensity physical activity (600 METs) per week [20]. Additionally, for men, having a waist circumference below 94 cm (37 in) was considered as a ‘low risk’, and above as a ‘high risk’ while for women, below 80 cm (31.5in) is low risk, and above as high risk.

### Covariates

Socio-demographic and economic characteristics such as age, sex, education, and marital status have proven to have some impact on the incidence of CVDs and were controlled for in exploring the relationship between hypertension, diabetes, and CVD. Table 1 presents the summary characteristics of the outcome variable (incidence of CVD), the two independent variables (hypertension and diabetes), and the co-variates considered in the model.

**Table 1.** Summary statistics of dependent, independent variables, and covariates.

Sociodemographic	
Sex	
Male	40.35 (1,841)
Female	59.65 (2,722)
Marital status	
Not married	61.56 (2,809)
Married	38.44 (1,754)
Employment status	
Employed	55.07 (2,513)
Not employed	44.93 (2,050)
Respondent age in years	41.39 ± 13.97
Education	7.80 ± 4.90
Ln (Individual income)	5.36 ± 5.06
***Chronic conditions***	
Hypertension status	
Yes (Positive)	51.72 (2,203)
Diabetes	
Yes (Positive)	6.05 (276)
Cardiovascular disease	
Yes (Positive)	10.21 (466)
***Recommended Lifestyle practices***	
Not smoking	
Yes	90.82 (4,144)
Unharmful alcohol Alcohol Consumption	
Yes	32.63 (1,489)
Adequate moderate physical activity	
Yes	24.52 (1,119)
Adequate fruit & vegetable consumption	
Yes	20.95 (3,607)

Values in the table represent %(n) or mean ± standard deviation.

## Estimation strategy

We explored a logit function for our primary model, where the incidence of CVDs was the outcome variable with hypertension and diabetes as the main independent variable alongside a vector of other explanatory variables, as specified in Equation (1):

(1) $$\mathrm{logit}\left(p\right)=\beta_{0}+\beta_{1}*\mathrm{NCD}+\;\beta_{2}\;*\;\mathrm{Age}+\beta_{3}*\mathrm{Sex}+\beta_{4}*\mathrm{Work}+\;\beta_{4}*\mathrm{Marital}\;\mathrm{status}+\;\beta_{4}*\ln\left(\mathrm{income}\right)$$

*p* is the probability of the event (incidence of CVD) and $\beta_{0}$, $\beta_{1}$, $\beta_{2}$, $\beta_{3}$, and $\beta_{4}$ are the coefficients associated with the intercept, hypertension/diabetes, age, marital status, formal education, working status, and log-transformed income, respectively. Beta estimates were selected over odds ratios in this empirical estimation to provide a more straightforward interpretation of the effect size and direction of the predictor variables than the odds ratio. Additionally, we provide marginal effects to show how a one-unit change in an independent variable affects the predicted probability of the outcome, making it easier to understand and explain.

The relationship between hypertension/diabetes and CVD may be subject to bias due to potential endogeneity arising from a bi-causal relationship between the dependent and independent variables. This reciprocal influence can distort the estimated coefficients, complicating the identification of the true effect of hypertension/diabetes on CVD incidence. Addressing this issue is essential. While instrumental variable (IV) methods are commonly employed to address endogeneity, we could not identify a suitable instrument that met the criteria for both relevance and exclusion restrictions. As a result, we employed the Kinky Least Squares (KLS) technique as an alternative method to address endogeneity-related biases [21]. This technique directly imposes bounds on the endogeneity correlation, limiting the correlation between the regressors and the error term to plausible levels. This approach removes the need for an instrumental variable and avoids the exclusion restriction requirement.

## Mediation analysis

We investigated the impact of residential location (urban vs. rural) on the relationship between living with either hypertension or diabetes and CVD among study respondents. This was aimed at identifying potential pathways in this association.

A sequential two-step methodology outlined by the subsequent equations was employed to examine the mediating effect of location in the hypertension-CVD nexus.

(2) $$\mathrm{Me}d_{i}=\omega_{0}+\omega_{1}\mathrm{Hypertension}/\mathrm{diabete}s_{i}+\sum_{k}^{\;}Y_{k}X_{k,i}+\beta_{c}+\varepsilon_{i}$$

(3) $$\mathrm{CV}D_{i}=\partial_{0}+\partial_{1}\mathrm{Hypertension}/\mathrm{diabete}s_{i}+\partial_{2}\mathrm{Me}d_{i}+\sum_{k}^{\;}Y_{k}X_{k,i}+\beta_{c}+\varepsilon_{i}$$

From Equation 2 above, $\mathrm{Me}d_{i}$ represents the mediating variable. For a variable to be considered a mediating variable, first, the co-efficient of hypertension/diabetes in equation 2 needs to be significant. Second, the inclusion of $\mathrm{Me}d_{i}$ equation 3 should render the co-efficient of hypertension/diabetes statistically insignificant (indicating complete mediation) or in a situation where $\partial_{1}$ is significant, $\mathrm{Me}d_{i}$ can be considered as partially mediating the relationship when $\partial_{1}$ is lesser than $\beta_{1}$ in equation 1.

## Moderation analysis

We investigated whether adherence to recommended lifestyle modifications could potentially weaken the extent to which hypertension influences the incidence of CVD. This relationship is mathematically demonstrated in the subsequent equation:

(4) $$\mathrm{CV}D_{i}=\beta_{0}+\frac{\beta_{1}\mathrm{Hypertension}}{\mathrm{diabetes}}+\beta_{2}\mathrm{Age}+\beta_{3}\mathrm{Sex}+\beta_{3}\mathrm{Married}+\beta_{4}\mathrm{Work}+\beta_{5}\mathrm{income}+\beta_{6}\mathrm{Hypertension}/\mathrm{diabetes}*\mathrm{ModV}+\beta_{7}\mathrm{ModV}+\varepsilon_{i}$$

In Equation 4, *ModV* represents adherence to various lifestyle modifications serving as moderating variable(s). Specifically, *ModV* represents recommendations about i) alcohol consumption, ii) fruit and vegetable intake, iii) engagement in physical activity, iv) keeping the recommended body weight (abdominal obesity), and v) a combination of all (i.e., alcohol consumption, fruits and vegetables, engagement in physical activity, and recommended abdominal fat). Thus, we concentrated on the effect of the interaction between hypertension and the specific lifestyle recommendation.

## Results

### Background characteristics

Table 1 presents the background characteristics of the study respondents. The sample comprised respondents with a mean age of 41.39 years (SD 13.97), and the majority (60%) were female. Approximately, 38% of the respondents were married. The average number of years of formal education was 7.80 (SD 4.90).

The prevalence of hypertension, diabetes, and CVD is 52%, 6%, and 10%, respectively. For lifestyle practices, 91% of the respondents reported not smoking within the past 30 days while 33% engaged in unharmed alcohol consumption. Only 24% of the respondents met the criteria for moderate physical activity and 21% consumed the recommended five or more servings of fruits and vegetables per day.

### Hypertension/Diabetes and CVD nexus

Table 2 presents logit results for 1) hypertension with the incidence of CVD and 2) diabetes with the incidence of CVD after controlling for other socio-demographic and economic characteristics.

**Table 2.** Association between hypertension, diabetes, and CVD.

		Hypertension				Diabetes			
		*Coeff.*	*Margins*		*RSE*	*Coeff.*	*Margins*		*RSE*
***B:*****Adjusted analyses**									
Hypertension/Diabetes (Yes)		0.458	**0.037**	***	0.118	1.263	0.110	***	0.141
			0.005	***					
Age		0.057	0.005	***	0.005	0.057	0.004	***	0.005
			0						
Female		0.784	0.064	***	0.126	0.712	0.057	****	0.125
Married		0.152	0.011		0.104	0.148	0.010	**	0.104
Education in years		−0.008	−0.001		0.012	−0.013	−0.001		0.013
Working (Yes)		−0.119	−0.010		0.114	−0.115	−0.010		0.115
Log (Household income)		0.004	0.000		0.003	0.003	0.000		0.011
Observations		4,563				4,563			
Wald chi2		290.2				348.1			
Pseudo R2		0.132				0.149			
***C: Kinky regression***									
Hypertension (Yes)		0.470		**	0.012	0.951		***	0.035
Control variables included		YES			YES				
Postulated endogeneity of hypertension		−0.500			−0.500				

***p < 0.01, **p < 0.05.

The F-statistics of both models confirm that for each model the variables collectively explain the incidence of CVD. This outcome aligns with our initial hypothesis (*H1*), which individuals with hypertension and those with diabetes are more likely to suffer from CVD. Results shown in the table (from the margin column) indicate that, for individuals with hypertension and diabetes, the probability of having CVD increases by 3.79% and 10.15% points, respectively.

Additionally, as a complimentary analysis of the preliminary results, we present results for hypertension/diabetes-CVD using propensity score matching (see Table 3) to verify whether the positive relationship will continue to exist when observables are controlled.

**Table 3.** Propensity scores match estimates for the effect of hypertension and diabetes on CVD.

	hypertension		diabetes	
Matching Technique	ATT	RSE	ATT	RSE
Nearest-neighbor matching	0.031***	0.011	0.112***	0.031
Regression adjustment matching	0.046***	0.009	0.101***	0.027
Augmented IPW	0.046***	0.009	0.101***	0.009
Observation	4,563		4,563	

***p<0.01.

The nearest-neighbor matching results show that hypertension and diabetes are associated with a 3.1% and 11.2% increase in the incidence of CVD, respectively. The estimates align with the variant matching algorithms (regression adjustment matching and augmented IPW). These results further buttress the association established which validates the robustness of our estimates.

### Mediation analysis

The results presented in Table 4 demonstrate a significant negative correlation between hypertension and living in an urban area. Specifically, at a 1% significance level, residing in an urban area is associated with a decreased likelihood of an individual having hypertension.

**Table 4.** Mediation effect of location in the hypertension/diabetes-CVD nexus.

	Living in an urban area	CVD
Hypertension/Diabetes (Yes)	−0.052***	0.055***
	(0.015)	(0.009)
Urban place (Yes)		0.028***
		(0.009)
Control variables included	Yes	Yes
**Diagnostics**		
RIT		0.028
Sobel test (z-value)		−0.002**
Observation	4,563	4,563

Robust standard errors in parentheses; ***p < 0.01, **p < 0.05.

To further validate this relationship and shed light on the mechanism through which hypertension/diabetes affects CVD, the Sobel test was conducted.

The Sobel test confirms that living in an urban area serves as a mediating factor through which hypertension and diabetes influence CVD. Approximately 3% of the effect of having either hypertension/diabetes on CVD is mediated by the location of the person (i.e., urban or rural). These findings substantiate the second hypothesis, affirming that residing in an urban area reduces the likelihood of developing hypertension/diabetes and consequently decreases the incidence of CVD.

From Table 5, individuals with hypertension or diabetes adhering to the suggested low-risk waist circumference (abdominal fat levels) were the only recommended lifestyle recommendation linked to a decreased risk of developing CVD. In other words, among individuals with high blood pressure or diabetes, sticking to the suggested waist size (which indicates low belly fat) was the only lifestyle practice tied to lower chances of having a CVD. The combined effect of the lifestyle modifications was tested; however, it was not significant.

**Table 5.** The moderating effect of lifestyle recommendations on the incidence of cardiovascular diseases in individuals with hypertension and diabetes.

Variables	Hypertension	Diabetes
*Interaction terms*	*Coeff. (RSE)*	*Coeff. (RSE)*
NCD & Smoking *(Smoking= 0, 1= Does not smoking)*	−0.12 (0.45)	0.43 (0.57)
NCD & Alcohol *(Consumes= 1, 0= Does not)*	0.03 (0.26)	0.00 (0.32)
NCD & Physical Activity *(Active = 1, 0= Not active)*	−0.33 (0.26)	0.01 (0.34)
NCD & Fruits & vegetables *(adequate= 1, 0= Not adequate)*	0.29 (0.27)	−0.29 (0.34)
NCD & Waist circumference *(Low risk= 1, 0= High risk)*	-0.50 (0.23)**	-0.82 (0.34)**
All Covariates	*Present*	*Present*

**p < 0.05: Standard errors in parentheses.

### Discussion

#### The silent partner

The connection between hypertension, diabetes, and the likelihood of CVD is confirmed after testing the first hypothesis. This relationship has been well-established in previous studies and is largely supported by the underlying biological mechanisms [22,23]. Both hypertension and/or diabetes may accelerate the buildup of plaques in the arteries, otherwise known as atherosclerosis, which has the potential of limiting the flow of blood to major organs in the body including the heart [24]. The buildup of plaques increases the likelihood of developing CVD. Generally, CVDs are often treated as standalone diseases. Results from this study add to existing scholarships that the occurrence of CVDs may be made worse by silent partners, hypertension, and diabetes. As the population of Cabo Verde continues to age, with a notable rise in NCDs such as hypertension and diabetes, management strategies must be put in place as these diseases are significant risk factors for CVD [25]. Conversely, given that hypertension was linked to an increased risk of CVD and all-cause mortality, with the associations being stronger for those with an earlier age of onset [26], it should be a concern not only for the elderly but among younger adults as well. Additionally, both diabetes and hypertension are recognized as opportunistic conditions that significantly contribute to mortality from other communicable and non-communicable diseases. For instance, Viswanathan et al. (2021) highlighted the impact of the diabetes-COVID-19 interaction on cardiovascular diseases (CVDs), showing how this loop can accelerate the onset of cardiovascular and cerebrovascular events [27].

#### The Urban-Rural conundrum

Cabo Verde is one of the most urbanized countries in the sub-Saharan Africa region. In just five decades, Cabo Verde’s urban population has skyrocketed from 20.6% in 1973 to a staggering 67.5% as of 2022. As simple as the relationship between residence and the incidence of hypertension/diabetes in CVD may look, the complexities involved tend to be multifaceted [28]. Whilst lifestyle choices in urban areas play a major role in this conundrum [29], it is important to highlight the key variable here, place of residence. In drawing the connection, pertinent factors such as the availability and accessibility of healthcare facilities are crucial. These are closely linked to income disparities, dietary choices, and environmental factors [30] bordering on the availability of green spaces and green belts to reduce both air and noise pollution, which are key components of the Social Determinants of Health (SDoH).

From the results, we assume that people with hypertension/diabetes living in urban areas have the advantage of having access to higher-level healthcare facilities for the treatment and management of their conditions, as purported by the SDoH. Again, the efforts made by Cabo Verde to strengthen its primary healthcare system using WHO PEN [25] are paying off. Since 2007, the MHSS of Cabo Verde has initiated the STEPS approach to examine the State’s chronic disease profile and risk factors. The adoption of healthy lifestyle choices, especially among urban residents, coupled with timely access to information on lifestyle modifications, otherwise unavailable to their counterparts living in rural areas, may serve as an advantage. The focus on implementing these lifestyle changes, as outlined in the SDoH, to reduce co-morbid conditions likely explains this outcome.

### Do lifestyle choices matter?

Lifestyle choices including diet, physical activity, smoking, and alcohol use as outlined by the World Health Organization to be modifiable behavioral risk factors of NCDs in general have been used to explain how at-risk populations further plunge themselves into NCD-related morbidity and mortality [31]. Testing the third hypothesis of this study revealed that in the current study sample, maintaining the recommended waist circumference moderated the established association between hypertension/diabetes and the incidence of CVD. Similarly, the association between waist circumference, which is often labeled as abdominal obesity, has been shown to increase the risk of CVD [32,33]. Thus, waist circumference is a required criterion for defining metabolic syndrome in any population. Abdominal obesity is seen as a stronger risk factor than overall obesity for CVD risk [34,35]. Persons with hypertension/diabetes are therefore encouraged to maintain the recommended waist circumference by engaging in physical activity, avoiding the abuse of alcohol, and adopting healthier eating habits. Although individually these lifestyle choices do not significantly affect the occurrence of CVD, their adoption is crucial in maintaining the recommended waist circumference. The simple answer then to whether lifestyle choices matter is yes.

## Limitations

Although the results are robust, the complex and long-term progression of non-communicable diseases (NCDs) and the evolving nature of social determinants of health suggest that a longitudinal dataset would be more appropriate. Such data can capture changes over time, providing deeper insights into how social factors continuously influence the development and outcomes of chronic diseases, which cannot be fully understood with a cross-sectional approach. Also, factors such as access to healthcare, genetics, and consumption of meat in high quantities, which are not observed in the data set, could potentially influence the conundrum between diabetes, hypertension, and other CVD. Additionally, the use of broad Urban and Rural categories in this study may oversimplify the complex variations within residential locations. To address this limitation, future research can introduce more detailed sub-categories and incorporate qualitative data to provide a richer understanding of how specific residential contexts influence the outcomes.

The above notwithstanding, the WHO STEPS survey employed a multi-stage cluster sample; thus, it is highly generalizable to the national population of the country in which it is conducted. While these limitations may exist, particularly regarding excluded populations or specific subpopulations, the robust sampling design and large sample sizes used generally ensure that the results can be extrapolated to the broader population with a high degree of confidence.

## Conclusion

Findings from this study could draw the attention of public health practitioners and policymakers, particularly in developing nations, to the varying dynamics place of residence plays in the incidence of non-communicable disease, in this case, urbanicity. This knowledge could improve the development of more effective policies and interventions specifically tailored to address CVD. This, in turn, holds the potential to significantly improve heart health outcomes and reduce the burden of this debilitating disease on individuals and healthcare systems globally.

## Data Availability

The datasets used and/or analyzed for the current study are available upon request at the World Health Organization website. The link is shown below. https://extranet.who.int/ncdsmicrodata/index.php/catalog/935
